# Supplementary material for: Experimental and observational studies find contrasting responses of soil nutrients to climate change
Source: eLife. 2017 Jun 1;6:e23255. doi: 10.7554/eLife.23255 (PMC5453695; doi:10.7554/eLife.23255)
Supplement: Supplementary file 1. — DOI: http://dx.doi.org/10.7554/eLife.23255.011 [file elife-23255-supp1.docx]

**These references in the supplementary word file are un-cited references for meta-analysis data.**

1. Abril A., Merlo C. & Noe L. (2013). Realistic soil C sink estimate in dry forests of western Argentina based on humic substance content. *Journal of Arid Environments*, 91, 113-118.
2. Alexander E.B. (2014). Arid to humid serpentine soils, mineralogy, and vegetation across the Klamath Mountains, USA. *CATENA*, 116, 114-122.
3. Allen K.W. (2012). Relationships between plant productivity and soil conditions in alpine tundra of southern Yukon Territory, Canada. In: *University of Saskatchewan*. University of Saskatchewan University of Saskatchewan.
4. Allison S.D., Czimczik C.I. & Treseder K.K. (2008). Microbial activity and soil respiration under nitrogen addition in Alaskan boreal forest. *Global Change Biology*, 14, 1156-1168.
5. Alvarez-Clare S. & Mack M.C. (2011). Influence of precipitation on soil and foliar nutrients across nine costa rican forests. *Biotropica*, 43, 433-441.
6. Amelung W. & Zech W. (1996). Organic species in ped surface and core fractions along a climosequence in the prairie, North America. *Geoderma*, 74, 193-206.
7. Anderson T.M., Ritchie M.E. & McNaughton S.J. (2007). Rainfall and soils modify plant community response to grazing in Serengeti National Park. *Ecology*, 88, 1191-1201.
8. Andresen L.C., Michelsen A., Ambus P. & Beier C. (2010). Belowground heathland responses after 2 years of combined warming, elevated CO_2_ and summer drought. *Biogeochemistry*, 101, 27-42.
9. Andresen L.C., Michelsen A., Jonasson S., Beier C. & Ambus P. (2009). Glycine uptake in heath plants and soil microbes responds to elevated temperature, CO_2_ and drought. *Acta Oecologica*, 35, 786-796.
10. Andruschkewitsch M., Wachendorf C., Sradnick A., Hensgen F., Joergensen R.G. & Wachendorf M. (2014). Soil substrate utilization pattern and relation of functional evenness of plant groups and soil microbial community in five low mountain NATURA 2000. *Plant and Soil*, 383, 275-289.
11. Angel R., Soares M.I.M., Ungar E.D. & Gillor O. (2010). Biogeography of soil archaea and bacteria along a steep precipitation gradient. *Isme Journal*, 4, 553-563.
12. Aranibar J.N., Otter L., Macko S.A., Feral C.J.W., Epstein H.E., Dowty P.R., Eckardt F., Shugart H.H. & Swap R.J. (2004). Nitrogen cycling in the soil-plant system along a precipitation gradient in the Kalahari sands. *Global Change Biology*, 10, 359-373.
13. Arnold J., Corre M.D. & Veldkamp E. (2009). Soil N cycling in old-growth forests across an Andosol toposequence in Ecuador. *Forest Ecology and Management*, 257, 2079-2087.
14. Austin A.T. & Vitousek P.M. (1998). Nutrient dynamics on a precipitation gradient in Hawai'i. *Oecologia*, 113, 519-529.
15. Austin A.T. (2002). Differential effects of precipitation on production and decomposition along a rainfall gradient in Hawaii. *Ecology*, 83, 328-338.
16. Bachar A., Al-Ashhab A., Soares M.I.M., Sklarz M.Y., Angel R., Ungar E.D. & Gillor O. (2010). Soil microbial abundance and diversity along a low precipitation gradient. *Microbial Ecology*, 60, 453-461.
17. Bahram M., Polme S., Koljalg U., Zarre S. & Tedersoo L. (2012). Regional and local patterns of ectomycorrhizal fungal diversity and community structure along an altitudinal gradient in the Hyrcanian forests of northern Iran. *New Phytologist*, 193, 465-473.
18. Barbhuiya A.R., Arunachalam A., Pandey H.N., Arunachalam K., Khan M.L. & Nath P.C. (2004). Dynamics of soil microbial biomass C, N and P in disturbed and undisturbed stands of a tropical wet-evergreen forest. *European Journal of Soil Biology*, 40, 113-121.
19. Barnard R., Barthes L., Le Roux X., Harmens H., Raschi A., Soussana J.F., Winkler B. & Leadley P.W. (2004). Atmospheric CO_2_ elevation has little effect on nitrifying and denitrifying enzyme activity in four European grasslands. *Global Change Biology*, 10, 488-497.
20. Barnard R., Le Roux X., Hungate B.A., Cleland E.E., Blankinship J.C., Barthes L. & Leadley P.W. (2006). Several components of global change alter nitrifying and denitrifying activities in an annual grassland. *Functional Ecology*, 20, 557-564.
21. Barrett J.E., Johnson D.W. & Burke I.C. (2002a). Abiotic nitrogen uptake in semiarid grassland soils of the US Great Plains. *Soil Science Society of America Journal*, 66, 979-987.
22. Barrett J.E., McCulley R.L., Lane D.R., Burke I.C. & Lauenroth W.K. (2002b). Influence of climate variability on plant production and N-mineralization in central US grasslands. *Journal of Vegetation Science*, 13, 383-394.
23. Bejarano M., Crosby M.M., Parra V., Etchevers J.D. & Campo J. (2014). Precipitation regime and nitrogen addition effects on leaf litter decomposition in tropical dry forests. *Biotropica*, 46, 415-424.
24. Belay-Tedla A., Zhou X.H., Su B., Wan S.Q. & Luo Y.Q. (2009). Labile, recalcitrant, and microbial carbon and nitrogen pools of a tallgrass prairie soil in the us great plains subjected to experimental warming and clipping. *Soil Biology & Biochemistry*, 41, 110-116.
25. Berger T.W., Duboc O., Djukic I., Tatzber M., Gerzabek M.H. & Zehetner F. (2015). Decomposition of beech (*Fagus sylvatica*) and pine (*Pinus nigra*) litter along an Alpine elevation gradient: Decay and nutrient release. *Geoderma*, 251–252, 92-104.
26. Bhattacharyya P., Roy K.S., Neogi S., Manna M.C., Adhya T.K., Rao K.S. & Nayak A.K. (2013). Influence of elevated carbon dioxide and temperature on belowground carbon allocation and enzyme activities in tropical flooded soil planted with rice. *Environmental Monitoring and Assessment*, 185, 8659-8671.
27. Bi J., Zhang N., Liang Y., Cai L. & Ma K. (2012). Impacts of increased N use and precipitation on microbial C utilization potential in the semiarid grassland of Inner Mongolia. *Chinese journal of eco-agriculture*, 20, 1586-1593.
28. Biasi C., Meyer H., Rusalimova O., Hammerle R., Kaiser C., Baranyi C., Daims H., Lashchinsky N., Barsukov P. & Richter A. (2008). Initial effects of experimental warming on carbon exchange rates, plant growth and microbial dynamics of a lichen-rich dwarf shrub tundra in Siberia. *Plant and Soil*, 307, 191-205.
29. Blankinship J.C., Brown J.R., Dijkstra P., Allwright M.C. & Hungate B.A. (2010). Response of Terrestrial CH_4_ Uptake to Interactive Changes in Precipitation and Temperature Along a Climatic Gradient. *Ecosystems*, 13, 1157-1170.
30. Bloor J.M.G. & Bardgett R.D. (2012). Stability of above-ground and below-ground processes to extreme drought in model grassland ecosystems: Interactions with plant species diversity and soil nitrogen availability. *Perspectives in Plant Ecology Evolution and Systematics*, 14, 193-204.
31. Bokhorst S., Bjerke J.W., Street L.E., Callaghan T.V. & Phoenix G.K. (2011). Impacts of multiple extreme winter warming events on sub-arctic heathland: Phenology, reproduction, growth, and CO_2_ flux responses. *Global Change Biology*, 17, 2817-2830.
32. Bokhorst S., Huiskes A., Convey P. & Aerts R. (2007). Climate change effects on organic matter decomposition rates in ecosystems from the Maritime Antarctic and Falkland Islands. *Global Change Biology*, 13, 2642-2653.
33. Bombonato L. & Gerdol R. (2012). Manipulating snow cover in an alpine bog: effects on ecosystem respiration and nutrient content in soil and microbes. *Climatic Change*, 114, 261-272.
34. Borghini F., Grimalt J.O., Sanchez-Hernandez J.C., Barra R., Garcia C.J.T. & Focardi S. (2005). Organochlorine compounds in soils and sediments of the mountain Andean Lakes. *Environmental Pollution*, 136, 253-266.
35. Bouskill N.J., Lim H.C., Borglin S., Salve R., Wood T.E., Silver W.L. & Brodie E.L. (2013). Pre-exposure to drought increases the resistance of tropical forest soil bacterial communities to extended drought. *Isme Journal*, 7, 384-394.
36. Brockett B.F.T., Prescott C.E. & Grayston S.J. (2012). Soil moisture is the major factor influencing microbial community structure and enzyme activities across seven biogeoclimatic zones in western Canada. *Soil Biology & Biochemistry*, 44, 9-20.
37. Buamscha G., Gobbi M., Mazzarino M.J. & Laos F. (1998). Indicators of nitrogen conservation in Austrocedrus chilensis forests along a moisture gradient in Argentina. *Forest Ecology and Management*, 112, 253-261.
38. Buckeridge K.M., Cen Y.P., Layzell D.B. & Grogan P. (2010). Soil biogeochemistry during the early spring in low arctic mesic tundra and the impacts of deepened snow and enhanced nitrogen availability. *Biogeochemistry*, 99, 127-141.
39. Butler S.M., Melillo J.M., Johnson J.E., Mohan J., Steudler P.A., Lux H., Burrows E., Smith R.M., Vario C.L., Scott L., Hill T.D., Aponte N. & Bowles F. (2012). Soil warming alters nitrogen cycling in a new england forest: Implications for ecosystem function and structure. *Oecologia*, 168, 819-828.
40. Cantarel A.A.M., Bloor J.M.G., Pommier T., Guillaumaud N., Moirot C., Soussana J.F. & Poly F. (2012). Four years of experimental climate change modifies the microbial drivers of N_2_O fluxes in an upland grassland ecosystem. *Global Change Biology*, 18, 2520-2531.
41. Chadwick O.A., Kelly E.F., Hotchkiss S.C. & Vitousek P.M. (2007). Precontact vegetation and soil nutrient status in the shadow of Kohala Volcano, Hawaii. *Geomorphology*, 89, 70-83.
42. Chapin F.S. (1981). Field-measurements of growth and phosphate absorption in *Carex aquatilis* along a latitudinal gradient. *Arctic and Alpine Research*, 13, 83-94.
43. Chen Z., Yin H., Wei Y. & Liu Q. (2010). Short-term effects of night warming and nitrogen addition on soil available nitrogen and microbial properties in subalpine coniferous forest, Western Sichuan, China. *Acta Phytoecologica Sinica*, 34, 1254-1264.
44. Cheng H., Ren W.W., Ding L.L., Liu Z.F. & Fang C.M. (2013). Responses of a rice-wheat rotation agroecosystem to experimental warming. *Ecological Research*, 28, 959-967.
45. Cheng X., Luo Y., Xu X., Sherry R. & Zhang Q. (2011). Soil organic matter dynamics in a north America tallgrass prairie after 9 yr of experimental warming. *Biogeosciences*, 8, 1487-1498.
46. Chimner R.A., Bonvissuto G.L., Victoria Cremona M., Gaitan J.J. & Lopez C.R. (2011). Ecohydrological conditions of wetlands along a precipitation gradient in Patagonia, Argentina. *Ecologia Austral*, 21, 329-337.
47. Christenson L.M., Mitchell M.J., Groffman P.M. & Lovett G.M. (2010). Winter climate change implications for decomposition in northeastern forests: comparisons of sugar maple litter with herbivore fecal inputs. *Global Change Biology*, 16, 2589-2601.
48. Cleveland C.C., Wieder W.R., Reed S.C. & Townsend A.R. (2010). Experimental drought in a tropical rain forest increases soil carbon dioxide losses to the atmosphere. *Ecology*, 91, 2313-2323.
49. Clinebell R.R., Phillips O.L., Gentry A.H., Stark N. & Zuuring H. (1995). Prediction of neotropical tree and liana species richness from soil and climatic data. *Biodiversity and Conservation*, 4, 56-90.
50. Collins S.L., Fargione J.E., Crenshaw C.L., Nonaka E., Elliott J.R., Xia Y. & Pockman W.T. (2010). Rapid plant community responses during the summer monsoon to nighttime warming in a northern Chihuahuan desert grassland. *Journal of Arid Environments*, 74, 611-617.
51. Contosta A.R., Frey S.D. & Cooper A.B. (2011). Seasonal dynamics of soil respiration and N mineralization in chronically warmed and fertilized soils. *Ecosphere*, 2, art36.
52. Corre M.D. & Lamersdorf N.P. (2004). Reversal of nitrogen saturation after long-term deposition reduction: Impact on soil nitrogen cycling. *Ecology*, 85, 3090-3104.
53. Couteaux M.M., Bottner P., Anderson J.M., Berg B., Bolger T., Casals P., Romanya J., Thiery J.M. & Vallejo V.R. (2001). Decomposition of C^13^-labelled standard plant material in a latitudinal transect of European coniferous forests: Differential impact of climate on the decomposition of soil organic matter compartments. *Biogeochemistry*, 54, 147-170.
54. Craft C., Washburn C. & Parker A. (2008). *Latitudinal trends in organic carbon accumulation in temperate freshwater peatlands*.
55. Craine J.M., Elmore A.J., Wang L.X., Augusto L., Baisden W.T., Brookshire E.N.J., Cramer M.D., Hasselquist N.J., Hobbie E.A., Kahmen A., Koba K., Kranabetter J.M., Mack M.C., Marin-Spiotta E., Mayor J.R., McLauchlan K.K., Michelsen A., Nardoto G.B., Oliveira R.S., Perakis S.S., Peri P.L., Quesada C.A., Richter A., Schipper L.A., Stevenson B.A., Tumer B.L., Viani R.A.G., Wanek W. & Zeller B. (2015). Convergence of soil nitrogen isotopes across global climate gradients. *Scientific Reports*, 5.
56. Cusack D.F., Torn M.S., McDowell W.H. & Silver W.L. (2010). The response of heterotrophic activity and carbon cycling to nitrogen additions and warming in two tropical soils. *Global Change Biology*, 16, 2555-2572.
57. Dai C., Kang M., Ji W. & Jiang Y. (2012a). Responses of belowground biomass and biomass allocation to environmental factors in central grassland of Inner Mongolia. *Acta Agrestia Sinica*, 20, 268-274.
58. Dai C., Kang M., Ji W. & Jiang Y. (2012b). Responses of underground productivity to biomass and environmental factors in Xilingol grassland, Inner Mongolia. *Chinese Journal of Grassland*, 34, 54-60.
59. Dai L., Li Y.Z., Luo G.P., Xu W.Q., Lu L., Li C.F. & Feng Y.X. (2013). The spatial variation of alpine timberlines and their biogeographical characteristics in the northern Tianshan Mountains of China. *Environmental Earth Sciences*, 68, 129-137.
60. Dawes M.A., Hagedorn F., Zumbrunn T., Handa I.T., Hattenschwiler S., Wipf S. & Rixen C. (2011). Growth and community responses of alpine dwarf shrubs to in situ CO_2_ enrichment and soil warming. *New Phytologist*, 191, 806-818.
61. Day T.A., Ruhland C.T. & Xiong F.S. (2008). Warming increases aboveground plant biomass and C stocks in vascular-plant-dominated antarctic tundra. *Global Change Biology*, 14, 1827-1843.
62. de Freitas A., de Sá Barretto Sampaio E., de Souza Ramos A., de Vasconcellos Barbosa M., Lyra R. & Araújo E. (2015). Nitrogen isotopic patterns in tropical forests along a rainfall gradient in Northeast Brazil. *Plant and Soil*, 1-14.
63. Delgado-Baquerizo M., Maestre F.T., Gallardol A., Bowker M.A., Wallenstein M.D., Luis Quero J., Ochoa V., Gozalo B., Garcia-Gomez M., Soliveres S., Garcia-Palacios P., Berdugo M., Valencia E., Escolar C., Arredondo T., Barraza-Zepeda C., Bran D., Antonio Carreiral J., Chaieb M., Conceicao A.A., Derak M., Eldridge D.J., Escudero A., Espinosa C.I., Gaitan J., Gatica M.G., Gomez-Gonzalez S., Guzman E., Gutierrez J.R., Florentino A., Hepper E., Hernandez R.M., Huber-Sannwald E., Jankju M., Liu J., Mau R.L., Miriti M., Monerris J., Naseri K., Noumi Z., Polo V., Prina A., Pucheta E., Ramirez E., Ramirez-Collantes D.A., Romao R., Tighe M., Torres D., Torres-Diaz C., Ungar E.D., Val J., Wamiti W., Wang D. & Zaady E. (2013). Decoupling of soil nutrient cycles as a function of aridity in global drylands. Nature, 502, 672-676.
64. DeMarco J., Mack M.C. & Bret-Harte M.S. (2011). The effects of snow, soil microenvironment, and soil organic matter quality on N availability in three Alaskan arctic plant communities. *Ecosystems*, 14, 804-817.
65. Dere A.L., White T.S., April R.H., Reynolds B., Miller T.E., Knapp E.P., McKay L.D. & Brantley S.L. (2013). Climate dependence of feldspar weathering in shale soils along a latitudinal gradient. *Geochimica et Cosmochimica Acta*, 122, 101-126.
66. Dijkstra F.A., Blumenthal D., Morgan J.A., Pendall E., Carrillo Y. & Follett R.F. (2010). Contrasting effects of elevated CO_2_ and warming on nitrogen cycling in a semiarid grassland. *New Phytologist*, 187, 426-437.
67. Dintwe K., Okin G., D’Odorico P., Hrast T., Mladenov N., Handorean A., Bhattachan A. & Caylor K.K. (2014). Soil organic C and total N pools in the Kalahari: potential impacts of climate change on C sequestration in savannas. *Plant and Soil*, 1-18.
68. Dong Y.S., Qi Y.C., Liu J.Y., Geng Y.B., Domroes M., Yang X.H. & Liu L.X. (2005). Variation characteristics of soil respiration fluxes in four types of grassland communities under different precipitation intensity. *Chinese Science Bulletin*, 50, 583-591.
69. Dormann C.F., van der Wal R. & Woodin S.J. (2004). Neighbour identity modifies effects of elevated temperature on plant performance in the high arctic. *Global Change Biology*, 10, 1587-1598.
70. Dou J.X., Liu J.S., Wang Y. & Zhao G.Y. (2010). Experimental soil-warming effects on carbon processes of typical meadow *Calamagrostis angustifolia* wetland ecosystem in the sanjiang plain, northeast China. *Acta Agriculturae Scandinavica Section B-Soil and Plant Science*, 60, 361-368.
71. Du B.M., Kang H.Z., Pumpanen J., Zhu P.H., Yin S., Zou Q., Wang Z., Kong F.Q. & Liu C.J. (2014). Soil organic carbon stock and chemical composition along an altitude gradient in the Lushan Mountain, subtropical China. *Ecological Research*, 29, 433-439.
72. Dubinsky E.A., Silver W.L. & Firestone M.K. (2010). Tropical forest soil microbial communities couple iron and carbon biogeochemistry. *Ecology*, 91, 2604-2612.
73. Duboc O., Zehetner F., Djukic I., Tatzber M., Berger T.W. & Gerzabek M.H. (2012). Decomposition of European beech and Black pine foliar litter along an Alpine elevation gradient: Mass loss and molecular characteristics. *Geoderma*, 189, 522-531.
74. Ebersberger D., Niklaus P.A. & Kandeler E. (2003). Long term CO_2_ enrichment stimulates N-mineralisation and enzyme activities in calcareous grassland. *Soil Biology & Biochemistry*, 35, 965-972.
75. Ebersberger D., Werrnbter N., Niklaus P.A. & Kandeler E. (2004). Effects of long term CO_2_ enrichment on microbial community structure in calcareous grassland. *Plant and Soil*, 264, 313-323.
76. Estop-Aragones C., Knorr K.H. & Blodau C. (2013). Belowground in situ redox dynamics and methanogenesis recovery in a degraded fen during dry-wet cycles and flooding. *Biogeosciences*, 10, 421-436.
77. Evans S.E. & Burke I.C. (2013). Carbon and nitrogen decoupling under an 11-year drought in the shortgrass steppe. *Ecosystems*, 16, 20-33.
78. Evans S.E. (2012). Microbial and biogeochemical responses to changing precipitation patterns in grassland ecosystems. In. COLORADO STATE UNIVERSITY.
79. Fajardo A. & Piper F.I. (2015). High foliar nutrient concentrations and resorption efficiency in *Embothrium coccineum (*Proteaceae) in southern Chile. *American Journal of Botany*, 102, 208-216.
80. Fang H., Cheng S., Wang Y., Yu G., Xu M., Dang X., Li L. & Wang L. (2014). Changes in soil heterotrophic respiration, carbon availability, and microbial function in seven forests along a climate gradient. *Ecological Research*, 29, 1077-1086.
81. Feng X.J., Simpson A.J., Wilson K.P., Williams D.D. & Simpson M.J. (2008). Increased cuticular carbon sequestration and lignin oxidation in response to soil warming. *Nature Geoscience*, 1, 836-839.
82. Fisher J.B., Malhi Y., Cuba Torres I., Metcalfe D.B., van de Weg M.J., Meir P., Silva-Espejo J.E. & Huaraca Huasco W. (2013). Nutrient limitation in rainforests and cloud forests along a 3,000-m elevation gradient in the Peruvian Andes. *Oecologia*, 172, 889-902.
83. Fitzhugh R.D., Driscoll C.T., Groffman P.M., Tierney G.L., Fahey T.J. & Hardy J.P. (2001). Effects of soil freezing disturbance on soil solution nitrogen, phosphorus, and carbon chemistry in a northern hardwood ecosystem. *Biogeochemistry*, 56, 215-238.
84. Fitzhugh R.D., Driscoll C.T., Groffman P.M., Tierney G.L., Fahey T.J. & Hardy J.P. (2003). Soil freezing and the acid-base chemistry of soil solutions in a northern hardwood forest. *Soil Science Society of America Journal*, 67, 1897-1908.
85. Fonseca C.R., Overton J.M., Collins B. & Westoby M. (2000). Shifts in trait-combinations along rainfall and phosphorus gradients. *Journal of Ecology*, 88, 964-977.
86. Fujimura K.E., Egger K.N. & Henry G.H. (2008). The effect of experimental warming on the root-associated fungal community of *Salix arctica*. *Isme Journal*, 2, 105-114.
87. Gavito M.E., Schweiger P. & Jakobsen I. (2003). P uptake by arbuscular mycorrhizal hyphae: Effect of soil temperature and atmospheric CO_2_ enrichment. *Global Change Biology*, 9, 106-116.
88. Gill R.A. (2014). The influence of 3-years of warming and N-deposition on ecosystem dynamics is small compared to past land use in subalpine meadows. *Plant and Soil*, 374, 197-210.
89. Gillison A. & Liswanti N. (1999). Impact of different land uses on biodiversity. In.
90. Girardin C.A.J., Aragao L.E.O.C., Malhi Y., Huaraca Huasco W., Metcalfe D.B., Durand L., Mamani M., Silva-Espejo J.E. & Whittaker R.J. (2013). Fine root dynamics along an elevational gradient in tropical Amazonian and Andean forests. *Global Biogeochemical Cycles*, 27, 252-264.
91. Gomez-Rey M.X., Madeira M., Gonzalez-Prieto S.J. & Coutinho J. (2010). Soil C and N dynamics within a precipitation gradient in Mediterranean eucalypt plantations. *Plant and Soil*, 336, 157-171.
92. Gornall J.L., Woodin S.J., Jonsdottir I.S. & van der Wal R. (2009). Herbivore impacts to the moss layer determine tundra ecosystem response to grazing and warming. *Oecologia*, 161, 747-758.
93. Harrington R.A., Fownes J.H., Meinzer F.C. & Scowcroft P.G. (1995). Forest Growth Along a Rainfall Gradient in Hawaii - Acacia-Koa Stand Structure, Productivity, Foliar Nutrients, and Water-Use and Nutrient-Use Efficiencies. *Oecologia*, 102, 277-284.
94. Haugwitz M.S., Bergmark L., Prieme A., Christensen S., Beier C. & Michelsen A. (2014). Soil microorganisms respond to five years of climate change manipulations and elevated atmospheric CO_2_ in a temperate heath ecosystem. *Plant and Soil*, 374, 211-222.
95. He J.S., Wang X.P., Schmid B., Flynn D.F.B., Li X.F., Reich P.B. & Fang J.Y. (2010). Taxonomic identity, phylogeny, climate and soil fertility as drivers of leaf traits across Chinese grassland biomes. *Journal of Plant Research*, 123, 551-561.
96. He M.Z. & Dijkstra F.A. (2015). Phosphorus addition enhances loss of nitrogen in a phosphorus-poor soil. *Soil Biology & Biochemistry*, 82, 99-106.
97. He N.P., Wang R.M., Zhang Y.H. & Chen Q.S. (2014). Carbon and nitrogen storage in Inner Mongolian grasslands: Relationships with climate and soil texture. *Pedosphere*, 24, 391-398.
98. Heng T., Wu J., Xie S. & Wu M. (2011). The responses of roil C and N, microbial biomass C or N under alpine meadow of Qinghai-Tibet Plateau to the change of temperature and precipitation. *Chinese Agricultural Science Bulletin*, 27, 425-430.
99. Hertel D., Strecker T., Mueller-Haubold H. & Leuschner C. (2013). Fine root biomass and dynamics in beech forests across a precipitation gradient - is optimal resource partitioning theory applicable to water-limited mature trees? *Journal of Ecology*, 101, 1183-2000.
100. Hinojosa M.B., Parra A., Ramirez D.A., Carreira J.A., Garcia-Ruiz R. & Moreno J.M. (2012). Effects of drought on soil phosphorus availability and fluxes in a burned Mediterranean shrubland. *Geoderma*, 191, 61-69.
101. Hobbie S.E. & Chapin F.S. (1998). An experimental test of limits to tree establishment in Arctic tundra. *Journal of Ecology*, 86, 449-461.
102. Hobbie S.E. (1996). Temperature and plant species control over litter decomposition in Alaskan tundra. *Ecological Monographs*, 66, 503-522.
103. Hollister E.B., Engledow A.S., Hammett A.J.M., Provin T.L., Wilkinson H.H. & Gentry T.J. (2010). Shifts in microbial community structure along an ecological gradient of hypersaline soils and sediments. *Isme Journal*, 4, 829-838.
104. Hood R.C. (2001). The effect of soil temperature and moisture on organic matter decomposition and plant growth. *Isotopes in Environmental and Health Studies*, 37, 25-41.
105. Hovenden M.J., Wills K.E., Chaplin R.E., Schoor J.K.V., Williams A.L., Osanai Y. & Newton P.C.D. (2008). Warming and elevated CO_2_ affect the relationship between seed mass, germinability and seedling growth in *Austrodanthonia caespitosa*, a dominant Australian grass. *Global Change Biology*, 14, 1633-1641.
106. Hu Z.-h., Zhou Y.-p., Cut H.-l., Chen S.-t., Xiao Q.-T. & Liu Y. (2013). Effects of diurnal warming on soil N_2_O emission in soybean field. *Huanjing Kexue*, 34, 2961-2967.
107. Huang X.-X., Yu Z.-Y., Qian W., Xu D.-Y. & Al G.-Y. (2007). Effects of adding water on seasonal variation of soil nitrogen availability under sandy grasslands in semi-arid region. *Journal of Forestry Research (Harbin)*, 18, 287-290.
108. Idol T., Baker P.J. & Meason D. (2007). Indicators of forest ecosystem productivity and nutrient status across precipitation and temperature gradients in Hawaii. *Journal of Tropical Ecology*, 23, 693-704.
109. Ippolito J.A., Blecker S.W., Freeman C.L., McCulley R.L., Blair J.M. & Kelly E.F. (2010). Phosphorus biogeochemistry across a precipitation gradient in grasslands of central North America. *Journal of Arid Environments*, 74, 954-961.
110. Ishizuka S., Kawamuro K., Imaya A., Torii A. & Morisada K. (2014). Latitudinal gradient of C-4 grass contribution to Black Soil organic carbon and correlation between delta C-13 and the melanic index in Japanese forest stands. *Biogeochemistry*, 118, 339-355.
111. James J.J. & Richards J.H. (2005). Plant N capture from pulses: effects of pulse size, growth rate, and other soil resources. *Oecologia*, 145, 113-122.
112. Jassey V.E.J., Chiapusio G., Binet P., Buttler A., Laggoun-Defarge F., Delarue F., Bernard N., Mitchell E.A.D., Toussaint M.L., Francez A.J. & Gilbert D. (2013). Above- and belowground linkages in *Sphagnum* peatland: climate warming affects plant-microbial interactions. *Global Change Biology*, 19, 811-823.
113. Jiang L., Zhu T., Ma L., Shi L., Hou W. & Guo J. (2011). Responses of ecosystem's carbon and water fluxes to global change on the Songnen Steppe. *Science & Technology Review*, 29, 35-42.
114. Jing X., Wang Y., Chung H., Mi Z., Wang S., Zeng H. & He J.-S. (2014). No temperature acclimation of soil extracellular enzymes to experimental warming in an alpine grassland ecosystem on the Tibetan Plateau. *Biogeochemistry*, 117, 39-54.
115. Johnson D.W., Hungate B.A., Dijkstra P., Hymus G. & Drake B. (2001). Effects of elevated carbon dioxide on soils in a Florida scrub oak ecosystem. *Journal of Environmental Quality*, 30, 501-507.
116. Jonasson S., Castro J. & Michelsen A. (2006). Interactions between plants, litter and microbes in cycling of nitrogen and phosphorus in the arctic. *Soil Biology & Biochemistry*, 38, 526-532.
117. Jonasson S., Havstrom M., Jensen M. & Callaghan T.V. (1993). In-situ mineralization of nitrogen and phosphorus of arctic soils after perturbations simulating climate-change. *Oecologia*, 95, 179-186.
118. Jones M.M., Simon F., Condit R., Manion G., Salomon A. & Pérez R. (2013). Strong congruence in tree and fern community turnover in response to soils and climate in central Panama. *Journal of Ecology*, 101, 506-516.
119. Jongen M., Lecomte X., Unger S., Pinto-Marijuan M. & Pereira J.S. (2013). The impact of changes in the timing of precipitation on the herbaceous understorey of Mediterranean evergreen oak woodlands. *Agricultural and Forest Meteorology*, 171, 163-173.
120. Kane E.S., Chivers M.R., Turetsky M.R., Treat C.C., Petersen D.G., Waldrop M., Harden J.W. & McGuire A.D. (2013). Response of anaerobic carbon cycling to water table manipulation in an Alaskan rich fen. *Soil Biology & Biochemistry*, 58, 50-60.
121. Kaste J.M., Bostick B.C., Heimsath A.M., Steinnes E. & Friedland A.J. (2011). Using atmospheric fallout to date organic horizon layers and quantify metal dynamics during decomposition. *Geochimica et Cosmochimica Acta*, 75, 1642-1661.
122. Khormali F. & Kehl M. (2011). Micromorphology and development of loess-derived surface and buried soils along a precipitation gradient in Northern Iran. *Quaternary International*, 234, 109-123.
123. Khormali F., Ghergherechi S., Kehl M. & Ayoubi S. (2012). Soil formation in loess-derived soils along a subhumid to humid climate gradient, Northeastern Iran. *Geoderma*, 179, 113-122.
124. Klose S., Wernecke K.D. & Makeschin F. (2003). Microbial biomass and enzyme activities in coniferous forest soils as affected by lignite-derived deposition. *Biology and Fertility of Soils*, 38, 32-44.
125. Kreyling J., Haei M. & Laudon H. (2012). Absence of snow cover reduces understory plant cover and alters plant community composition in boreal forests. *Oecologia*, 168, 577-587.
126. Krywult M., Smykla J., Kinnunen H., Martz F., Sutinen M.L., Lakkala K. & Turunen M. (2008). Influence of solar uv radiation on the nitrogen metabolism in needles of Scots pine (*Pinus sylvestris* L.). *Environmental Pollution*, 156, 1105-1111.
127. Kuster T.M., Arend M., Bleuler P., Gunthardt-Goerg M.S. & Schulin R. (2013). Water regime and growth of young oak stands subjected to air-warming and drought on two different forest soils in a model ecosystem experiment. *Plant Biology*, 15, 138-147.
128. Lai L.M., Li Y.F., Tian Y., Jiang L.H., Zhao X.C., Zhu L.H., Chen X., Gao Y., Wang S.M., Zheng Y.R. & Rimmington G.M. (2013). Effects of added organic matter and water on soil carbon sequestration in an arid region. *Plos One*, 8.
129. Landesman W.J. & Dighton J. (2010). Response of soil microbial communities and the production of plant-available nitrogen to a two-year rainfall manipulation in the New Jersey Pinelands. *Soil Biology & Biochemistry*, 42, 1751-1758.
130. Lang S.I., Cornelissen J.H.C., Holzer A., ter Braak C.J.F., Ahrens M., Callaghan T.V. & Aerts R. (2009). Determinants of cryptogam composition and diversity in *Sphagnum*-dominated peatlands: the importance of temporal, spatial and functional scales. *Journal of Ecology*, 97, 299-310.
131. Larionova A.A., Kurganova I.N., de Gerenyu V.O.L., Zolotareva B.N., Yevdokimov I.V. & Kudeyarov V.N. (2010). Carbon dioxide emissions from agrogray soils under climate changes. *Eurasian Soil Science*, 43, 168-176.
132. Larsen K.S., Andresen L.C., Beier C., Jonasson S., Albert K.R., Ambus P., Arndal M.F., Carter M.S., Christensen S., Holmstrup M., Ibrom A., Kongstad J., van der Linden L., Maraldo K., Michelsen A., Mikkelsen T.N., Pilegaard K., Prieme A., Ro-Poulsen H., Schmidt I.K., Selsted M.B. & Stevnbak K. (2011). Reduced N cycling in response to elevated CO_2_, warming, and drought in a danish heathland: Synthesizing results of the climaite project after two years of treatments. *Global Change Biology*, 17, 1884-1899.
133. Lawrence D. (2005). Regional-scale variation in litter production and seasonality in tropical dry forests of southern Mexico. *Biotropica*, 37, 561-570.
134. Leffler A.J. & Welker J.M. (2013). Long-term increases in snow pack elevate leaf N and photosynthesis in *Salix arctica*: responses to a snow fence experiment in the High Arctic of NW Greenland. *Environmental Research Letters*, 8.
135. Lehman R.M., Garland J.L. & Osborne S.L. (2013). Applying an oxygen-based respiratory assay to assess soil microbial responses to substrate and N availability. *Applied Soil Ecology*, 64, 127-134.
136. Lemenih M. & Itanna F. (2004). Soil carbon stocks and turnovers in various vegetation types and arable lands along an elevation gradient in southern Ethiopia. *Geoderma*, 123, 177-188.
137. Li H., Ye D.D., Wang X.G., Settles M.L., Wang J., Hao Z.Q., Zhou L.S., Dong P., Jiang Y. & Ma Z.S. (2014a). Soil bacterial communities of different natural forest types in Northeast China. *Plant and Soil*, 383, 203-216.
138. Li N., Wang G.X., Gao Y.H. & Wang J.F. (2011a). Warming effects on plant growth, soil nutrients, microbial biomass and soil enzymes activities of two alpine meadows in Tibetan Plateau. *Polish Journal of Ecology*, 59, 25-35.
139. Li N., Wang G.X., Yang Y., Gao Y.H. & Liu G.S. (2011b). Plant production, and carbon and nitrogen source pools, are strongly intensified by experimental warming in alpine ecosystems in the Qinghai-Tibet plateau. *Soil Biology & Biochemistry*, 43, 942-953.
140. Li P., Yang Y.H., Han W.X. & Fang J.Y. (2014b). Global patterns of soil microbial nitrogen and phosphorus stoichiometry in forest ecosystems. *Global Ecology and Biogeography*, 23, 979-987.
141. Li Q., Bai H., Liang W., Xia J., Wan S. & van der Putten W.H. (2013a). Nitrogen addition and warming independently influence the belowground micro-food web in a temperate steppe. *Plos One*, 8, e60441-e60441.
142. Li W., Bai E., Li S.-l., Sun J.-f., Peng B. & Jiang P. (2013b). Effects of nitrogen addition and precipitation change on soil methane and carbon dioxide fluxes. *Shengtaixue Zazhi*, 32, 1947-1958.
143. Li X.Z. & Chen Z.Z. (2004). Soil microbial biomass C and N along a climatic transect in the Mongolian steppe. *Biology and Fertility of Soils*, 39, 344-351.
144. Liu M., Liu G., Gong L., Wang D. & Sun J. (2014). Relationships of biomass with environmental factors in the grassland area of Hulunbuir, China. *Plos One*, 9.
145. Liu Q., Yin H.J., Chen J.S., Zhao C.Z., Cheng X.Y., Wei Y.Y. & Lin B. (2011). Belowground responses of *Picea asperata* seedlings to warming and nitrogen fertilization in the eastern Tibetan Plateau. *Ecological Research*, 26, 637-648.
146. Liu X.M., Li Q., Liang W.J. & Jiang Y. (2008a). Distribution of soil enzyme activities and microbial biomass along a latitudinal gradient in farmlands of Songliao Plain, Northeast China. *Pedosphere*, 18, 431-440.
147. Liu X.R., Dong Y.S., Ren J.Q. & Li S.G. (2010). Drivers of soil net nitrogen mineralization in the temperate grasslands in Inner Mongolia, China. *Nutrient Cycling in Agroecosystems*, 87, 59-69.
148. Liu Y., Chen S., Liu Y., Gao H., An J., Zhen X., Li H. & Wang M. (2013). Effects of simulated warming on the key processes of soil carbon and nitrogen cycling in a cropland. *China Environmental Science*, 33, 674-679.
149. Liu Y.S., Pan Q.M., Zheng S.X., Bai Y.F. & Han X.G. (2012). Intra-seasonal precipitation amount and pattern differentially affect primary production of two dominant species of Inner Mongolia grassland. *Acta Oecologica-International Journal of Ecology*, 44, 2-10.
150. Liu Z.F., Liu G.H., Fu B.J. & Zheng X.X. (2008b). Relationship between plant species diversity and soil microbial functional diversity along a longitudinal gradient in temperate grasslands of Hulunbeir, Inner Mongolia, China. *Ecological Research*, 23, 511-518.
151. Lü X.T. & Han X.G. (2010). Nutrient resorption responses to water and nitrogen amendment in semi-arid grassland of Inner Mongolia, China. *Plant and Soil*, 327, 481-491.
152. Lü X.T., Kong D.L., Pan Q.M., Simmons M.E. & Han X.G. (2012). Nitrogen and water availability interact to affect leaf stoichiometry in a semi-arid grassland. *Oecologia*, 168, 301-310.
153. Luo T.X., Zhang L., Zhu H.Z., Daly C., Li M.C. & Luo J. (2009). Correlations between net primary productivity and foliar carbon isotope ratio across a Tibetan ecosystem transect. *Ecography*, 32, 526-538.
154. Ma L., Guo C., Xin X., Yuan S. & Wang R. (2013). Effects of belowground litter addition, increased precipitation and clipping on soil carbon and nitrogen mineralization in a temperate steppe. *Biogeosciences*, 10, 7361-7372.
155. Ma L.N., Lu X.T., Liu Y., Guo J.X., Zhang N.Y., Yang J.Q. & Wang R.Z. (2011). The effects of warming and nitrogen addition on soil nitrogen cycling in a temperate grassland, northeastern China. *Plos One*, 6.
156. Ma Y. & Shen W. (2013). Facility effects on soil nitrogen transformation rates in a field controlled precipitation pattern change experiment. *Journal of Tropical and Subtropical Botany*, 21, 505-513.
157. Macrae M.L., Devito K.J., Strack M. & Waddington J.M. (2013). Effect of water table drawdown on peatland nutrient dynamics: implications for climate change. *Biogeochemistry*, 112, 661-676.
158. Malchair S., De Boeck H.J., Lemmens C., Merckx R., Nijs I., Ceulemans R. & Carnol M. (2010). Do climate warming and plant species richness affect potential nitrification, basal respiration and ammonia-oxidizing bacteria in experimental grasslands? *Soil Biology & Biochemistry*, 42, 1944-1951.
159. Marcos G.M. & Lancho J.F.G. (2002). Atmospheric deposition in oligotrophic *Quercus pyrenaica* forests: implications for forest nutrition. *Forest Ecology and Management*, 171, 17-29.
160. Matson P.A., Gower S.T., Volkmann C., Billow C. & Grier C.C. (1992). Soil nitrogen cycling and nitrous oxide flux in a Rocky. Mountain Douglas-fir forest: Effects of fertilization, irrigation and carbon addition. *Biogeochemistry*, 18, 101-117.
161. McCulley R.L., Burke I.C., Nelson J.A., Lauenroth W.K., Knapp A.K. & Kelly E.F. (2005). Regional patterns in carbon cycling across the great plains of north America. *Ecosystems*, 8, 106-121.
162. McDaniel M.D., Kaye J.P. & Kaye M.W. (2013). Increased temperature and precipitation had limited effects on soil extracellular enzyme activities in a post-harvest forest. *Soil Biology & Biochemistry*, 56, 90-98.
163. Menge D.N.L. & Field C.B. (2007). Simulated global changes alter phosphorus demand in annual grassland. *Global Change Biology*, 13, 2582-2591.
164. Mercader J., Bennett T., Esselmont C., Simpson S. & Walde D. (2011). Soil phytoliths from miombo woodlands in Mozambique. *Quaternary Research*, 75, 138-150.
165. Meyer H., Kaiser C., Biasi C., Hammerle R., Rusalimova O., Lashchinsky N., Baranyi C., Daims H., Barsukov P. & Richter A. (2006). Soil carbon and nitrogen dynamics along a latitudinal transect in western siberia, russia. *Biogeochemistry*, 81, 239-252.
166. Milla R., Castro-Diez P., Maestro-Martinez M. & Montserrat-Marti G. (2005). Does the gradualness of leaf shedding govern nutrient resorption from senescing leaves in Mediterranean woody plants? *Plant and Soil*, 278, 303-313.
167. Miranda J. (2008). Cambio climático y patrones de precipitación: efecto sobre las comunidades vegetales semiáridas. *Ecosistemas*, 17, 161-165.
168. Monokrousos N., Papatheodorou E.M. & Stamou G.P. (2011). Under climatic change, soil microbial community and variables relating to N-cycle are modulated by changes in the upper limit temperature. *Global Nest Journal*, 13, 385-394.
169. Moreno G., Gallardo J.F. & Bussotti F. (2001). Canopy modification of atmospheric deposition in oligotrophic *Quercus pyrenaica* forests of an unpolluted region (central-western Spain). *Forest Ecology and Management*, 149, 47-60.
170. Moreno G., Gallardo J.F., Schneider K. & Ingelmo F. (1996). Water and bioelement fluxes in four Quercus pyrenaica forests along a pluviometric gradient. *Annales Des Sciences Forestieres*, 53, 625-639.
171. Mueller-Haubold H., Hertel D., Seidel D., Knutzen F. & Leuschner C. (2013). Climate responses of aboveground productivity and allocation in Fagus sylvatica: A transect study in mature forests. *Ecosystems*, 16, 1498-1516.
172. Nemergut D.R., Cleveland C.C., Wieder W.R., Washenberger C.L. & Townsend A.R. (2010). Plot-scale manipulations of organic matter inputs to soils correlate with shifts in microbial community composition in a lowland tropical rain forest. *Soil Biology & Biochemistry*, 42, 2153-2160.
173. Nguyen Xuan Que V. & Kang H. (2013). Regulation of soil enzyme activities in constructed wetlands under a short-term drying period. *Chemistry and Ecology*, 29, 146-165.
174. Niklaus P.A., Alphei D., Ebersberger D., Kampichler C., Kandeler E. & Tscherko D. (2003). Six years of in situ CO_2_ enrichment evoke changes in soil structure and soil biota of nutrient-poor grassland. *Global Change Biology*, 9, 585-600.
175. Niklaus P.A., Alphei J., Kampichler C., Kandeler E., Korner C., Tscherko D. & Wohlfender M. (2007). Interactive effects of plant species diversity and elevated CO_2_ on soil biota and nutrient cycling. *Ecology*, 88, 3153-3163.
176. Niu S.L., Sherry R.A., Zhou X.H., Wan S.Q. & Luo Y.Q. (2010). Nitrogen regulation of the climate-carbon feedback: Evidence from a long-term global change experiment. *Ecology*, 91, 3261-3273.
177. Nottingham A.T., Turner B.L., Stott A.W. & Tanner E.V.J. (2015). Nitrogen and phosphorus constrain labile and stable carbon turnover in lowland tropical forest soils. *Soil Biology & Biochemistry*, 80, 26-33.
178. O'Halloran L.R., Shugart H.H., Wang L.X., Caylor K.K., Ringrose S. & Kgope B. (2010). Nutrient limitations on aboveground grass production in four savanna types along the Kalahari transect. *Journal of Arid Environments*, 74, 284-290.
179. Ostonen I., Lohmus K., Helmisaari H.S., Truu J. & Meel S. (2007). Fine root morphological adaptations in Scots pine, Norway spruce and silver birch along a latitudinal gradient in boreal forests. *Tree Physiology*, 27, 1627-1634.
180. Owliaie H. (2014). A magnetic investigation along a NE-SW transect of the Yasouj Plain, southwestern Iran. *Archives of Agronomy and Soil Science*, 60, 1015-1027.
181. Ozolincius R., Stakenas V., Varnagiryte-Kabasinskiene I. & Buozyte R. (2009). Artificial drought in Scots pine stands: effects on soil, ground vegetation and tree condition. *Annales Botanici Fennici*, 46, 299-307.
182. Pan X., Li N.B. & Liu Q. (2008). Effects of elevated temperature on soil organic carbon and soil respiration under subalpine coniferous forest in western Sichuan Province,China. *Chinese Journal of Applied Ecology*, 19, 1637-1643.
183. Parsons S.A., Lawler I.R., Congdon R.A. & Williams S.E. (2011). Rainforest litter quality and chemical controls on leaf decomposition with near-infrared spectrometry. *Journal of Plant Nutrition and Soil Science*, 174, 710-720.
184. Pellicer J., Estiarte M., Garcia S., Garnatje T., Penuelas J., Sardans J. & Valles J. (2010). Genome size unaffected by moderate changes in climate and phosphorus availability in Mediterranean plants. *African Journal of Biotechnology*, 9, 6070-6077.
185. Pereira E.I.P., Chung H., Scow K. & Six J. (2013). Microbial communities and soil structure are affected by reduced precipitation, but not by elevated carbon dioxide. *Soil Science Society of America Journal*, 77, 482-488.
186. Perez-Harguindeguy N., Diaz S., Vendramini F., Gurvich D.E., Cingolani A.M., Giorgis M.A. & Cabido M. (2007). Direct and indirect effects of climate on decomposition in native ecosystems from central Argentina. *Austral Ecology*, 32, 749-757.
187. Peri P.L., Ladd B., Pepper D.A., Bonser S.P., Laffan S.W. & Amelung W. (2012). Carbon ^13^C and nitrogen ^15^N stable isotope composition in plant and soil in Southern Patagonia's native forests. *Global Change Biology*, 18, 311-321.
188. Piper F.I., Baeza G., Zuniga-Feest A. & Fajardo A. (2013). Soil nitrogen, and not phosphorus, promotes cluster-root formation in a South American Proteaceae, *Embothrium coccineum*. *American Journal of Botany*, 100, 2328-2338.
189. Porder S. & Chadwick O.A. (2009). Climate and soil-age constraints on nutrient uplift and retention by plants. *Ecology*, 90, 623-636.
190. Propster J. & Johnson N. (2015). Uncoupling the effects of phosphorus and precipitation on arbuscular mycorrhizas in the Serengeti. *Plant and Soil*, 388, 21-34.
191. Pugnaire F.I., Zhang L., Li R. & Luo T. (2015). No evidence of facilitation collapse in the Tibetan plateau. *Journal of Vegetation Science*, 26, 233-242.
192. Raich J.W., Russell A.E. & Vitousek P.M. (1997). Primary productivity and ecosystem development along an elevational gradient on Mauna Loa, Hawai'i. *Ecology*, 78, 707-721.
193. Ramirez K.S., Craine J.M. & Fierer N. (2012). Consistent effects of nitrogen amendments on soil microbial communities and processes across biomes. *Global Change Biology*, 18, 1918-1927.
194. Rappa M., Reginab I.S., Ricob M. & Gallegob H.A. (1999). Biomass, nutrient content, litterfall and nutrient return to the soil in Mediterranean oak forests. *Forest Ecology and Management*, 119, 39-49.
195. Ren Y. (2012). Effects of precipitation change on inorganic nitrogen and net nitrogen mineralization rate at a plantation of Mongolian pine. *Acta Scientiarum Naturalium Universitatis Pekinensis*, 48, 925-932.
196. Reynolds J.F., Virginia R.A., Kemp P.R., de Soyza A.G. & Tremmel D.C. (1999). Impact of drought on desert shrubs: Effects of seasonality and degree of resource island development. *Ecological Monographs*, 69, 69-106.
197. Rinnan R. & Rinnan A. (2007). Application of near infrared reflectance (nir) and fluorescence spectroscopy to analysis of microbiological and chemical properties of arctic soil. *Soil Biology & Biochemistry*, 39, 1664-1673.
198. Rinnan R., Michelsen A., Baath E. & Jonasson S. (2007a). Fifteen years of climate change manipulations alter soil microbial communities in a subarctic heath ecosystem. *Global Change Biology*, 13, 28-39.
199. Rinnan R., Michelsen A., Baath E. & Jonasson S. (2007b). Mineralization and carbon turnover in subarctic heath soil as affected by warming and additional litter. *Soil Biology & Biochemistry*, 39, 3014-3023.
200. Rinnan R., Stark S. & Tolvanen A. (2009). Responses of vegetation and soil microbial communities to warming and simulated herbivory in a subarctic heath. *Journal of Ecology*, 97, 788-800.
201. Roa-Fuentes L.L., Campo J. & Parra-Tabla V. (2012). Plant Biomass Allocation across a Precipitation Gradient: An Approach to Seasonally Dry Tropical Forest at Yucatan, Mexico. *Ecosystems*, 15, 1234-1244.
202. Robroek B.J.M., Heijboer A., Jassey V.E.J., Hefting M.M., Rouwenhorst T.G., Buttler A. & Bragazza L. (2013). Snow cover manipulation effects on microbial community structure and soil chemistry in a mountain bog. *Plant and Soil*, 369, 151-164.
203. Rogers M.C., Sullivan P.F. & Welker J.M. (2011). Evidence of nonlinearity in the response of net ecosystem CO_2_ exchange to increasing levels of winter snow depth in the high arctic of northwest Greenland. *Arctic Antarctic and Alpine Research*, 43, 95-106.
204. Rousk J., Frey S.D. & Baath E. (2012). Temperature adaptation of bacterial communities in experimentally warmed forest soils. *Global Change Biology*, 18, 3252-3258.
205. Routhier M.C. & Lapointe L. (2002). Impact of tree leaf phenology on growth rates and reproduction in the spring flowering species *Trillium erectum* (Liliaceae). *American Journal of Botany*, 89, 500-505.
206. Ruan W.B., Sang Y., Chen Q., Zhu X., Lin S. & Gao Y.B. (2012). The response of soil nematode community to nitrogen, water, and grazing history in the Inner Mongolian steppe, China. *Ecosystems*, 15, 1121-1133.
207. Rui Y.C., Wang S.P., Xu Z.H., Wang Y.F., Chen C.R., Zhou X.Q., Kang X.M., Lu S.B., Hu Y.G., Lin Q.Y. & Luo C.Y. (2011). Warming and grazing affect soil labile carbon and nitrogen pools differently in an alpine meadow of the Qinghai-Tibet plateau in China. *Journal of Soils and Sediments*, 11, 903-914.
208. Ryan J., Masri S. & Garabet S. (1996). Geographical distribution of soil test values in Syria and their relationship with crop response. *Communications in Soil Science and Plant Analysis*, 27, 1579-1593.
209. Sager E.P.S. & Hutchinson T.C. (2005). The effects of UV-B, nitrogen fertilization, and springtime warming on sugar maple seedlings and the soil chemistry of two central Ontario forests. *Canadian Journal of Forest Research*, 35, 2432-2446.
210. Sagova-Mareckova M., Omelka M., Cermak L., Kamenik Z., Olsovska J., Hackl E., Kopecky J. & Hadacek F. (2011). Microbial communities show parallels at sites with distinct litter and soil characteristics. *Applied and Environmental Microbiology*, 77, 7560-7567.
211. Saiz G., Bird M.I., Domingues T., Schrodt F., Schwarz M., Feldpausch T.R., Veenendaal E., Djagbletey G., Hien F., Compaore H., Diallo A. & Lloyd J. (2012). Variation in soil carbon stocks and their determinants across a precipitation gradient in West Africa. *Global Change Biology*, 18, 1670-1683.
212. Salinas-Peba L., Parra-Tabla V., Campo J. & Munguia-Rosas M.A. (2014). Survival and growth of dominant tree seedlings in seasonally tropical dry forests of Yucatan: site and fertilization effects. *Journal of Plant Ecology*, 7, 470-479.
213. Santiago L.S. (2003). Leaf traits of canopy trees on a precipitation gradient in Panama: integrating plant physiological ecology and ecosystem science. In. UNIVERSITY OF FLORIDA.
214. Santiago L.S., Schuur E.A.G. & Silvera K. (2005). Nutrient cycling and plant-soil feedbacks along a precipitation gradient in lowland panama. *Journal of Tropical Ecology*, 21, 461-470.
215. Sardans J. & Penuelas J. (2007). Drought changes phosphorus and potassium accumulation patterns in an evergreen Mediterranean forest. *Functional Ecology*, 21, 191-201.
216. Sardans J., Penuelas J. & Estiarte M. (2006). Warming and drought alter soil phosphatase activity and soil P availability in a Mediterranean shrubland. *Plant and Soil*, 289, 227-238.
217. Sardans J., Penuelas J. & Estiarte M. (2008a). Changes in soil enzymes related to C and N cycle and in soil C and N content under prolonged warming and drought in a Mediterranean shrubland. *Applied Soil Ecology*, 39, 223-235.
218. Sardans J., Penuelas J. & Ogaya R. (2008c). Drought-induced changes in C and N stoichiometry in a *Quercus ilex* Mediterranean forest. *Forest Science*, 54, 513-522.
219. Sardans J., Penuelas J. & Ogaya R. (2008d). Experimental drought reduced acid and alkaline phosphatase activity and increased organic extractable P in soil in a *Quercus ilex* Mediterranean forest. *European Journal of Soil Biology*, 44, 509-520.
220. Sardans J., Penuelas J., Estiarte M. & Prieto P. (2008b). Warming and drought alter C and N concentration, allocation and accumulation in a Mediterranean shrubland. *Global Change Biology*, 14, 2304-2316.
221. Sardans J., Penuelas J., Prieto P. & Estiarte M. (2008e). Drought and warming induced changes in P and k concentration and accumulation in plant biomass and soil in a Mediterranean shrubland. *Plant and Soil*, 306, 261-271.
222. Satti P., Mazzarino M.J., Roselli L. & Crego P. (2007). Factors affecting soil P dynamics in temperate volcanic soils of southern Argentina. *Geoderma*, 139, 229-240.
223. Schaeffer S.M., Sharp E., Schimel J.P. & Welker J.M. (2013). Soil-plant N processes in a High Arctic ecosystem, NW Greenland are altered by long-term experimental warming and higher rainfall. *Global Change Biology*, 19, 3529-3539.
224. Scheer C., Grace P.R., Rowlings D.W. & Payero J. (2013). Soil N_2_O and CO_2_ emissions from cotton in Australia under varying irrigation management. *Nutrient Cycling in Agroecosystems*, 95, 43-56.
225. Schindlbacher A., Rodler A., Kuffner M., Kitzler B., Sessitsch A. &Zechmeister-Boltenstern S. (2011). Experimental warming effects on the microbial community of a temperate mountain forest soil. *Soil Biology & Biochemistry*, 43, 1417-1425.
226. Schindlbacher A., Zechmeister-Boltenstern S., Kitzler B. & Jandl R. (2008). Experimental forest soil warming: response of autotrophic and heterotrophic soil respiration to a short-term 10 degrees C temperature rise. *Plant and Soil*, 303, 323-330.
227. Schuur E.A.G., Chadwick O.A. & Matson P.A. (2001). Carbon cycling and soil carbon storage in mesic to wet Hawaiian montane forests. *Ecology*, 82, 3182-3196.
228. Shaw M.R. & Harte J. (2001). Response of nitrogen cycling to simulated climate change: Differential responses along a subalpine ecotone. *Global Change Biology*, 7, 193-210.
229. Sheng W.P., Ren S.J., Yu G.R., Fang H.J., Jiang C.M. & Zhang M. (2011). Patterns and driving factors of WUE and NUE in natural forest ecosystems along the north-south transect of eastern China. *Journal of Geographical Sciences*, 21, 651-665.
230. Shi F.S., Chen H., Chen H.F., Wu Y. & Wu N. (2012). The combined effects of warming and drying suppress CO_2_ and N_2_O emission rates in an alpine meadow of the eastern Tibetan Plateau. *Ecological Research*, 27, 725-733.
231. Shi L.-L., Mortimer P.E., Slik J.W.F., Zou X.-M., Xu J., Feng W.-T. & Qiao L. (2014). Variation in forest soil fungal diversity along a latitudinal gradient. *Fungal Diversity*, 64, 305-315.
232. Singh J.S., Singh D.P. & Kashyap A.K. (2010). Microbial biomass C, N and P in disturbed dry tropical forest soils, India. *Pedosphere*, 20, 780-788.
233. Sistla S.A., Moore J.C., Simpson R.T., Gough L., Shaver G.R. & Schimel J.P. (2013). Long-term warming restructures Arctic tundra without changing net soil carbon storage. *Nature*, 497, 615-+.
234. Smolander A., Barnette L., Kitunen V. & Lumme I. (2005). N and C transformations in long-term N-fertilized forest soils in response to seasonal drought. *Applied Soil Ecology*, 29, 225-235.
235. Song M.H., Dong M. & Jiang G.M. (2002). Importance of clonal plants and plant species diversity in the Northeast China Transect. *Ecological Research*, 17, 705-716.
236. Sorensen P.L. & Michelsen A. (2011). Long-term warming and litter addition affects nitrogen fixation in a subarctic heath. *Global Change Biology*, 17, 528-537.
237. Sorensen P.L., Michelsen A. & Jonasson S. (2008). Nitrogen uptake during one year in subarctic plant functional groups and in microbes after long-term warming and fertilization. *Ecosystems*, 11, 1223-1233.
238. Souto C.P., Premoli A.C. & Reich P.B. (2009). Complex bioclimatic and soil gradients shape leaf trait variation in Embothrium coccineum (Proteaceae) among austral forests in Patagonia. *Revista Chilena de Historia Natural*, 82, 209-222.
239. Steinweg J.M., Fisk M.C., McAlexander B., Groffman P.M. & Hardy J.P. (2008). Experimental snowpack reduction alters organic matter and net N mineralization potential of soil macroaggregates in a northern hardwood forest. *Biology and Fertility of Soils*, 45, 1-10.
240. Sun X.M., Zhang X.K., Zhang S.X., Dai G.H., Han S.J. & Liang W.J. (2013). Soil nematode responses to increases in nitrogen deposition and precipitation in a temperate forest. *Plos One*, 8.
241. Tang L., Han W., Chen Y. & Fang J. (2013). Resorption proficiency and efficiency of leaf nutrients in woody plants in eastern China. *Journal of Plant Ecology*, 6, 408-417.
242. Tataw J.T., Hall R., Ziss E., Schwarz T., Buchwald C.V.U., Formayer H., Hosch J., Baumgarten A., Berthold H., Michel K. & Zaller J.G. (2014). Soil types will alter the response of arable agroecosystems to future rainfall patterns. *Annals of Applied Biology*, 164, 35-45.
243. Thomas A.D., Hoon S.R. & Dougill A.J. (2011). Soil respiration at five sites along the Kalahari Transect: Effects of temperature, precipitation pulses and biological soil crust cover. *Geoderma*, 167-68, 284-294.
244. Thomsen I.K., Laegdsmand M. & Olesen J.E. (2010). Crop growth and nitrogen turnover under increased temperatures and low autumn and winter light intensity. *Agriculture Ecosystems & Environment*, 139, 187-194.
245. Touchette B.W. & Burkholder J.M. (2007). Effects of temperature and nitrate on phosphomonoesterase activities between carbon source and sink tissues in *Zostera marina* L. *Journal of Experimental Marine Biology and Ecology*, 342, 313-324.
246. Tremolada P., Villa S., Bazzarin P., Bizzotto E., Comolli R. & Vighi M. (2008). POPs in mountain soils from the Alps and Andes: Suggestions for a 'precipitation effect' on altitudinal gradients. *Water Air and Soil Pollution*, 188, 93-109.
247. Truax B., Gagnon D., Fortier J. & Lambert F. (2012). Yield in 8 year-old hybrid poplar plantations on abandoned farmland along climatic and soil fertility gradients. *Forest Ecology and Management*, 267, 228-239.
248. Tscherko D., Kandeler E. & Jones T.H. (2001). Effect of temperature on below-ground N-dynamics in a weedy model ecosystem at ambient and elevated atmospheric CO_2_ levels. *Soil Biology & Biochemistry*, 33, 491-501.
249. Turner B.L. & Engelbrecht B.M.J. (2011). Soil organic phosphorus in lowland tropical rain forests. *Biogeochemistry*, 103, 297-315.
250. Turner M.M. & Henry H.A.L. (2010). Net nitrogen mineralization and leaching in response to warming and nitrogen deposition in a temperate old field: The importance of winter temperature. *Oecologia*, 162, 227-236.
251. Turrion M.-B., Schneider K. & Gallardo J.F. (2008). Soil P availability along a catena located at the Sierra de Gata Mountains, Western Central Spain. *Forest Ecology and Management*, 255, 3254-3262.
252. Turrion M.B., Schneider K. & Gallardo J.F. (2009). Carbon accumulation in Umbrisols under *Quercus pyrenaica* forests: Effects of bedrock and annual precipitation. *CATENA*, 79, 1-8.
253. Updegraff K., Pastor J., Bridgham S.D. & Johnston C.A. (1995). Environmental and substrate controls over carbon and nitrogen mineralization in northern wetlands. *Ecological Applications*, 5, 151-163.
254. van Breemen N., Jenkins A., Wright R.F., Beerling D.J., Arp W.J., Berendse F., Beier C., Collins R., Van Dam D., Rasmussen L., Verburg P.S.J. & Wills M.A. (1998). Impacts of elevated carbon dioxide and temperature on a boreal forest ecosystem (CLIMEX project). *Ecosystems*, 1, 345-351.
255. van Breugel M., Hall J.S., Craven D.J., Gregoire T.G., Park A., Dent D.H., Wishnie M.H., Mariscal E., Deago J., Ibarra D., Cedeno N. & Ashton M.S. (2011). Early growth and survival of 49 tropical tree species across sites differing in soil fertility and rainfall in Panama. *Forest Ecology and Management*, 261, 1580-1589.
256. van Straaten O., Veldkamp E., Kohler M. & Anas I. (2010). Spatial and temporal effects of drought on soil CO_2_ efflux in a cacao agroforestry system in Sulawesi, Indonesia. *Biogeosciences*, 7, 1223-1235.
257. Verburg P.S.J. & van Breemen N. (2000). Nitrogen transformations in a forested catchment in southern Norway subjected to elevated temperature and CO_2_. *Forest Ecology and Management*, 129, 31-39.
258. Verburg P.S.J. (2005). Soil solution and extractable soil nitrogen response to climate change in two boreal forest ecosystems. *Biology and Fertility of Soils*, 41, 257-261.
259. Verburg P.S.J., Johnson D.W., Schorran D.E., Wallace L.L., Luo Y. & Arnone J.A. (2009). Impacts of an anomalously warm year on soil nitrogen availability in experimentally manipulated intact tallgrass prairie ecosystems. *Global Change Biology*, 15, 888-900.
260. Verburg P.S.J., Van Loon W.K.P. & Lukewille A. (1999). The climex soil-heating experiment: Soil response after 2 years of treatment. *Biology and Fertility of Soils*, 28, 271-276.
261. Vitousek P.M. & Chadwick O.A. (2013). Pedogenic thresholds and soil process domains in basalt-derived soils. *Ecosystems*, 16, 1379-1395.
262. Wan S.Q., Hui D.F., Wallace L. & Luo Y.Q. (2005). Direct and indirect effects of experimental warming on ecosystem carbon processes in a tallgrass prairie. *Global Biogeochemical Cycles*, 19.
263. Wang C.H., Wan S.Q., Xing X.R., Zhang L. & Han X.G. (2006). Temperature and soil moisture interactively affected soil net N mineralization in temperate grassland in northern China. *Soil Biology & Biochemistry*, 38, 1101-1110.
264. Wang C.T., Long R.J., Wang Q.L., Jing Z.C. & Shi J.J. (2009). Changes in plant diversity, biomass and soil C, in alpine meadows at different degradation stages in the headwater region of Three Rivers, China. *Land Degradation & Development*, 20, 187-198.
265. Wang G.X., Ran F., Chang R.Y., Yang Y., Luo J. & Fan J.R. (2014a). Variations in the live biomass and carbon pools of Abies georgei along an elevation gradient on the Tibetan Plateau, China. *Forest Ecology and Management*, 329, 255-263.
266. Wang J.Y., Song C.C., Wang X.W. & Song Y.Y. (2012). Changes in labile soil organic carbon fractions in wetland ecosystems along a latitudinal gradient in Northeast China. *CATENA*, 96, 83-89.
267. Wang L., D'Odorico P., Ringrose S., Coetzee S. & Macka S.A. (2007). Biogeochemistry of Kalahari sands. *Journal of Arid Environments*, 71, 259-279.
268. Wang L., Zhang Y., Wang Z., Tu Y. & Shang E. (2013). Effects of grazing and hydrological disturbance on soil properties of wet meadow wetland in Lhasa. *Research of Soil and Water Conservation*, 20, 66-69.
269. Wang R.L., Yu G.R., He N.P., Wang Q.F., Xia F.C., Zhao N., Xu Z.W. & Ge J.P. (2014b). Elevation-related variation in leaf stomatal traits as a function of plant functional type: evidence from Changbai Mountain, China. *Plos One*, 9.
270. Wang S., Zhou G., Gao S. & Guo J. (2005a). Gradient distribution of soil nitrogen and its response to climate change along the Northeast China Transect. *Ying yong sheng tai xue bao*, 16, 279-83.
271. Wang S.P., Zhou G.S., Gao S.H. & Guo J.P. (2005b). Soil organic carbon and labile carbon along a precipitation gradient and their responses to some environmental changes. *Pedosphere*, 15, 676-680.
272. Wei H., Wu B., Yang W. & Luo T. (2011). Low rainfall-induced shift in leaf trait relationship within species along a semi-arid sandy land transect in northern China. *Plant Biology*, 13, 85-92.
273. Wei J.-m., Jiang Y., Fu M.-m., Zhang Y.-g. & Xu Z.-w. (2011). Effects of water addition and fertilization on soil nutrient contents and pH value of typical grassland in Inner Mongolia. *Shengtaixue Zazhi*, 30, 1642-1646.
274. Welker J.M., Fahnestock J.T., Henry G.H.R., O'Dea K.W. & Chimner R.A. (2004). CO_2_ exchange in three Canadian high arctic ecosystems: Response to long-term experimental warming. *Global Change Biology*, 10, 1981-1995.
275. Wichern F. & Joergensen R.G. (2009). Soil microbial properties along a precipitation transect in southern Africa. *Arid Land Research and Management*, 23, 115-126.
276. Wieder W.R., Cleveland C.C. & Townsend A.R. (2011). Throughfall exclusion and leaf litter addition drive higher rates of soil nitrous oxide emissions from a lowland wet tropical forest. *Global Change Biology*, 17, 3195-3207.
277. Williams M.A., Rice C.W. & Owensby C.E. (2001). Nitrogen competition in a tallgrass prairie ecosystem exposed to elevated carbon dioxide. *Soil Science Society of America Journal*, 65, 340-346.
278. Wright I.J. & Westoby M. (2002). Leaves at low versus high rainfall: coordination of structure, lifespan and physiology. *New Phytologist*, 155, 403-416.
279. Wu G.-L., Ren G.-H., Wang D., Shi Z.-H. & Warrington D. (2013). Above- and below-ground response to soil water change in an alpine wetland ecosystem on the Qinghai-Tibetan Plateau, China. *Journal of Hydrology*, 476, 120-127.
280. Wu X., Yao Z., Brueggemann N., Shen Z.Y., Wolf B., Dannenmann M., Zheng X. & Butterbach-Bahl K. (2010). Effects of soil moisture and temperature on CO_2_ and CH_4_ soil atmosphere exchange of various land use/cover types in a semi-arid grassland in Inner Mongolia, China. *Soil Biology & Biochemistry*, 42, 773-787.
281. Wu X.W., Duffy J.E., Reich P.B. & Sun S.C. (2011). A brown-world cascade in the dung decomposer food web of an alpine meadow: effects of predator interactions and warming. *Ecological Monographs*, 81, 313-328.
282. Xiong P., Xu Z., Lin B. & Liu Q. (2010). Short-term response of winter soil respiration to simulated warming in a *Pinus armandii* plantation in the upper reaches of the Minjiang River, China. *Acta Phytoecologica Sinica*, 34, 1369-1376.
283. Xiong Y.M., Zeng H., Xia H.P. & Guo D.L. (2014). Interactions between leaf litter and soil organic matter on carbon and nitrogen mineralization in six forest litter-soil systems. *Plant and Soil*, 379, 217-229.
284. Xu Z.F., Hu R., Xiong P., Wan C.A., Cao G. & Liu Q. (2010a). Initial soil responses to experimental warming in two contrasting forest ecosystems, eastern Tibetan Plateau, China: Nutrient availabilities, microbial properties and enzyme activities. *Applied Soil Ecology*, 46, 291-299.
285. Xu Z.F., Wan C.A., Xiong P., Tang Z., Hu R., Cao G. & Liu Q. (2010b). Initial responses of soil CO_2_ efflux and C, N pools to experimental warming in two contrasting forest ecosystems, eastern Tibetan Plateau, China. *Plant and Soil*, 336, 183-195.
286. Xu Z.F., Yin H.J., Xiong P., Wan C. & Liu Q. (2012). Short-term responses of *Picea asperata* seedlings of different ages grown in two contrasting forest ecosystems to experimental warming. *Environmental and Experimental Botany*, 77, 1-11.
287. Yahdjian L. & Sala O.E. (2008). Do litter decomposition and nitrogen mineralization show the same trend in the response to dry and wet years in the Patagonian steppe? *Journal of Arid Environments*, 72, 687-695.
288. Yahdjian L., Sala O. & Austin A.T. (2006). Differential controls of water input on litter decomposition and nitrogen dynamics in the Patagonian steppe. *Ecosystems*, 9, 128-141.
289. Yang H.S., Yuan Y.G., Zhang Q., Tang J.J., Liu Y. & Chen X. (2011). Changes in soil organic carbon, total nitrogen, and abundance of arbuscular mycorrhizal fungi along a large-scale aridity gradient. *CATENA*, 87, 70-77.
290. Yang W., Zheng Y., Gao C., He X.H., Ding Q., Kim Y., Rui Y.C., Wang S.P. & Guo L.D. (2013a). The arbuscular mycorrhizal fungal community response to warming and grazing differs between soil and roots on the Qinghai-Tibetan Plateau. *Plos One*, 8.
291. Yang Y., Li C., Zhang Y. & Cui Y. (2013b). Effects of submergence and drought alternation on nutrient contents in the soil growing slash pine (*Pinus elliottii*) seedlings. *Scientia Silvae Sinicae*, 49, 55-65.
292. Yano Y., Shaver G.R., Rastetter E.B., Giblin A.E. & Laundre J.A. (2013). Nitrogen dynamics in arctic tundra soils of varying age: differential responses to fertilization and warming. *Oecologia*, 173, 1575-1586.
293. Yao F.Y., Chen Y.H., Yan Z.B., Li P., Han W.X. & Fang J.Y. (2015). Biogeographic patterns of structural traits and C:N:P stoichiometry of tree twigs in China's forests. *Plos One*, 10.
294. Yavitt J.B., Wieder R.K. & Wright S.J. (1993). Soil nutrient dynamics in response to irrigation of a Panamanian tropical moist forest. *Biogeochemistry*, 19, 1-25.
295. Yergeau E., Bokhorst S., Kang S., Zhou J.Z., Greer C.W., Aerts R. & Kowalchuk G.A. (2012). Shifts in soil microorganisms in response to warming are consistent across a range of antarctic environments. *Isme Journal*, 6, 692-702.
296. Yin H.J., Li Y.F., Xiao J., Xu Z.F., Cheng X.Y. & Liu Q. (2013a). Enhanced root exudation stimulates soil nitrogen transformations in a subalpine coniferous forest under experimental warming. *Global Change Biology*, 19, 2158-2167.
297. Yin H.J., Xiao J., Li Y.F., Chen Z., Cheng X.Y., Zhao C.Z. & Liu Q. (2013b). Warming effects on root morphological and physiological traits: The potential consequences on soil C dynamics as altered root exudation. *Agricultural and Forest Meteorology*, 180, 287-296.
298. Yin H.J., Xu Z.F., Chen Z., Wei Y.Y. & Liu Q. (2012). Nitrogen transformation in the rhizospheres of two subalpine coniferous species under experimental warming. *Applied Soil Ecology*, 59, 60-67.
299. Yu Q., Epstein H.E., Walker D.A., Frost G.V. & Forbes B.C. (2011). Modeling dynamics of tundra plant communities on the Yamal Peninsula, Russia, in response to climate change and grazing pressure. *Environmental Research Letters*, 6, e04505.
300. Yuan Y.L., Si G.C., Wang J., Luo T.X. & Zhang G.X. (2014). Bacterial community in alpine grasslands along an altitudinal gradient on the Tibetan Plateau. *FEMS Microbiology Ecology*, 87, 121-132.
301. Yuste J.C., Ma S. & Baldocchi D.D. (2010). Plant-soil interactions and acclimation to temperature of microbial-mediated soil respiration may affect predictions of soil CO_2_ efflux. *Biogeochemistry*, 98, 127-138.
302. Zak D.R., Pregitzer K.S., Curtis P.S., Teeri J.A., Fogel R. & Randlett D.L. (1993). Elevated atmospheric CO_2_ and feedback between carbon and nitrogen cycles. *Plant and Soil*, 151, 105-117.
303. Zelikova T.J., Housman D.C., Grote E.E., Neher D.A. & Belnap J. (2012). Warming and increased precipitation frequency on the colorado plateau: Implications for biological soil crusts and soil processes. *Plant and Soil*, 355, 265-282.
304. Zhang B., Liang C., He H. & Zhang X. (2013). Variations in Soil Microbial Communities and Residues Along an Altitude Gradient on the Northern Slope of Changbai Mountain, China. *Plos One*, 8.
305. Zhang K., Johnson L., Yuan W., Pei Z., Chang S.I. & Wang D. (2014a). Glucan Yield from Enzymatic Hydrolysis of Big Bluestem as Affected by Ecotype and Planting Location Along the Precipitation Gradient of the Great Plains. *Bioenergy Research*, 7, 799-810.
306. Zhang M., Chen J., Guo J., Tian Y., Yang S., Zhang L., Yang B. & Zhang W. (2013a). Effects of nighttime warming on winter wheat root growth and soil nutrient availability. *Chinese Journal of Applied Ecology*, 24, 445-450.
307. Zhang N., Guo R., Song P., Guo J. & Gao Y. (2013b). Effects of warming and nitrogen deposition on the coupling mechanism between soil nitrogen and phosphorus in Songnen Meadow Steppe, northeastern China. *Soil Biology & Biochemistry*, 65, 96-104.
308. Zhang W., Parker K.M., Luo Y., Wan S., Wallace L.L. & Hu S. (2005). Soil microbial responses to experimental warming and clipping in a tallgrass prairie. *Global Change Biology*, 11, 266-277.
309. Zhang X., Liu W., Schloter M., Zhang G., Chen Q., Huang J., Li L., Elser J.J. & Han X. (2013c). Response of the abundance of key soil microbial nitrogen-cycling genes to multi-factorial global changes. *Plos One*, 8, e76500-e76500.
310. Zhang X.Y., Wang W., Chen W.L., Zhang N.L. & Zeng H. (2014b). Comparison of seasonal soil microbial process in snow-covered temperate ecosystems of northern China. *Plos One*, 9.
311. Zheng Y., Yang W., Sun X., Wang S.P., Rui Y.C., Luo C.Y. & Guo L.D. (2012). Methanotrophic community structure and activity under warming and grazing of alpine meadow on the Tibetan Plateau. *Applied Microbiology and Biotechnology*, 93, 2193-2203.
312. Zhou G., Wang Y. & Wang S. (2002). Responses of grassland ecosystems to precipitation and land use along the Northeast China Transect. *Journal of Vegetation Science*, 13, 361-368.
313. Zhou L., Song M.H., Wang S.Q., Fan J.W., Liu J.Y., Zhong H.P., Yu G.R., Gao L.P., Hu Z.M., Chen B., Wu W.X. & Song T. (2014). Patterns of soil ^15^N and total N and their relationships with environmental factors on the Qinghai-Tibetan Plateau. *Pedosphere*, 24, 232-242.
314. Zhou X., Chen C., Wang Y., Xu Z., Han H., Li L. & Wan S. (2013a). Warming and increased precipitation have differential effects on soil extracellular enzyme activities in a temperate grassland. *Science of the Total Environment*, 444, 552-558.
315. Zhou X., Chen C., Wang Y., Xu Z., Hu Z., Cui X. & Hao Y. (2012). Effects of warming and increased precipitation on soil carbon mineralization in an Inner Mongolian grassland after 6 years of treatments. *Biology and Fertility of Soils*, 48, 859-866.
316. Zhou X.Q., Chen C.R., Wang Y.F., Xu Z.H., Duan J.C., Hao Y.B. & Smaill S. (2013b). Soil extractable carbon and nitrogen, microbial biomass and microbial metabolic activity in response to warming and increased precipitation in a semiarid Inner Mongolian grassland. *Geoderma*, 206, 24-31.
317. Zhou Y.-D., Chen S.-P., Song W.-M., Lu Q. & Lin G.-H. (2011). Water-use strategies of two desert plants along a precipitation gradient in northwestern China. *Acta Phytoecologica Sinica*, 35, 789-800.
318. Zhou Y.M., Tang J.W., Melillo J.M., Butler S. & Mohan J.E. (2011). Root standing crop and chemistry after six years of soil warming in a temperate forest. *Tree Physiology*, 31, 707-717.
319. Zong N., Shi P., Jiang J., Song M., Xiong D., Ma W., Fu G., Zhang X. & Shen Z. (2013). Responses of ecosystem CO_2_ fluxes to short-term experimental warming and nitrogen enrichment in an alpine meadow, northern Tibet Plateau. *Scientific World Journal*.
320. Zou Y., Han F., Geng L. & Shen Y. (2010). Effects of temperature and moisture on soil nitrogen mineralization of lucerne stands. *Acta Prataculturae Sinica*, 19, 101-107.
